# Left Ventricular Mechanical Support with the Impella during Extracorporeal Membrane Oxygenation

**Published:** 2017-01

**Authors:** Kasra Moazzami, Elena V Dolmatova, Thomas P Cocke, Elie Elmann, Pranay Vaidya, Arthur F Ng, Kumar Satya, Rajeev L Narayan

**Affiliations:** 1 *Hackensack University Medical Center, Hackesack, NJ, USA. *; 2 *Rutgers New Jersey Medical School, Newark, NJ, USA.*; 3 *Valley Medical Group and Valley Hospital, Ridgewood, NJ, USA.*

**Keywords:** *Heart-assist devices*, *Extracorporeal membrane oxygenation*, *Heart ventricles*, *Treatment outcome*

## Abstract

**Background: **Venoarterial extracorporeal membrane oxygenation (ECMO) provides systemic arterial support without directly unloading the left heart, which causes an elevated left ventricular (LV) pressure. The aim of the present study was to investigate the adjunctive application of the Impella device for LV unloading in patients during ECMO.

**Methods:** This retrospective cohort study included patients who received Impella support in addition to venoarterial ECMO between April 2012 and December 2015. ECMO cannulation was performed peripherally or centrally, while the Impella device was surgically inserted into the femoral artery or the right axillary artery.

**Results: **Among 62 patients, 10 (16.1%) received an Impella device during ECMO support. Following Impella support, right atrial pressure improved from a median of 18 (IQR, 14–24) mmHg to 13 (IQR, 10–15) mmHg and pulmonary wedge pressure improved from 30 (IQR, 26–35) mmHg to 16 (IQR, 12–19) mmHg in all the patients (p value < 0.001). Follow-up transthoracic echocardiograms (n = 6) showed a median decrease of 0.8 cm in LV end-diastolic volume (p value = 0.021). There were 5 (50%) in-hospital deaths due to sustained brain injury (n = 3) and refractory cardiogenic shock (n = 2). The remaining 5 patients were discharged and successfully bridged to more permanent LV assist device (n = 2) or heart transplantation (n = 3).

**Conclusion:** The findings of the present study indicate that the application of the Impella device during ECMO support is effective in LV unloading and confers optimal hemodynamic support.

## Introduction

Venoarterial extracorporeal membrane oxygenation (ECMO) as a device for mechanical circulatory support was initially proposed in the early 1960s.^   1 ^ Over the past decade, its use has resurged in the adult population, with studies indicating its efficacy for various etiologies of cardiac arrest as well as in patients with refractory cardiogenic shock .^2^^, ^^3^ ECMO presents major advantages including feasibility of implantation, easy access, and cost-effectiveness compared to other circulatory devices.^4^^, ^^5^ However, ECMO provides systemic arterial support without directly unloading the left heart. Therefore, despite the advantages of ECMO support, the inadequate unloading of the left ventricle (LV) could cause an elevated LV pressure.^6^^-^^8^ This in turn can increase myocardial wall stress and result in ischemia, delayed ventricular recovery, and eventually pulmonary venous hypertension and pulmonary edema.^9^^, ^^10^ 

The Impella device is a catheter-mounted microaxial pump capable of providing a continuous blood flow by draining it from the LV and delivering it to the aortic root.^11^^-^^13^ The device has been shown to provide short-term circulatory support by augmenting the cardiac output and unloading the LV in postcardiotomic heart failure or refractory cardiogenic shock settings.^        14^

The purpose of the present study was to present the results of the adjunctive application of the Impella device for LV unloading in patients during ECMO support. 

## Methods

In this retrospective cohort study, between April 2012 and December 2015, all patients who received venoarterial ECMO support in conjunction with Impella support during the same hospitalization at the Hackensack University Medical Center were recruited. Baseline characteristics including age, gender, body mass index, associated comorbidities, baseline ejection fraction, indications for ECMO and Impella support, and the total duration of ECMO duration were collected. An informed consent was obtained from all the patients. The study was approved by the institutional review board at the Hackensack University Medical Center. 

Peripheral venoarterial ECMO (MAQUET Cardiopulmonary AG, Hirrlingen, Germany) was established through the femoral artery and the femoral vein under fluoroscopy guidance. The arterial cannula was placed in the distal aorta, proximal to the iliac bifurcation. The tip of the venous cannula was placed at the junction of the inferior vena cava and the right atrium. In addition, central ECMO cannulation was performed by using the left and right atria for access and positioning the outflow cannula into the ascending aorta. 

The Impella 2.5 device (ABIOMED Europe GmbH, Aachen, Germany) was surgically inserted into the femoral artery or into the right axillary artery. The device was positioned across the aortic valve into the LV under the guidance of both fluoroscopy and transesophageal ultrasound. 

All the data were analyzed using SPSS, version 18.0 (SPSS Inc., Chicago, IL, USA). The χ^2^ test and the Mann–Whitney test were used for the categorical and continuous variables, respectively. All the continuous variables are presented as medians (interquartile ranges). A p value < 0.05 was considered significant.

## Results

During the study period, a total of 62 patients received venoarterial ECMO support. Impella support was required in 10 (16.1%) patients. The median age of the cohort was 59 years (43–68), and 70.0% were male. All the baseline characteristics are summarized in Table 1. The indications for ECMO and Impella support comprised shock post myocardial infarction in 4 patients, postcardiotomic cardiogenic shock in 3 patients, cardiogenic shock in the setting of chronic heart failure in 2 patients, and cardiogenic shock secondary to primary donor graft failure in 1 patient. Peripheral and central ECMO was established in 8 and 2 patients, respectively. The median duration of Impella and ECMO support was 1 (1–2.5) day and 6.5 (2–11.3) days, correspondingly. Among the survivors, the median duration of Impella support was 3 (2–5) days. Five patients received mechanical circulatory support via the intra-aortic balloon pump prior to ECMO and Impella support. Hypertension and coronary artery disease were the most common associated comorbidities in the entire cohort (70.0%) (Table 1). In 9 patients, hemodynamic stability was achieved from mechanical support provided by Impella and ECMO combination. Immediately following Impella support, right atrial pressure improved from a median of 18 (14–24) mmHg to 13 (10–15) mmHg and pulmonary wedge pressure improved from 30 (26–35) mmHg to 16 (12–19) mmHg in all the patients (p value < 0.001). Also, follow-up transthoracic echocardiography was done in 6 patients and showed a median decrease of 0.8 cm in LV end-diastolic volume (p value = 0.021). 

There were 5 in-hospital deaths. No differences existed between the survivors and non-survivors in the baseline characteristics (Table 1), except for the incidence of chronic kidney disease. Among the non-survivors, 60.0% had a history of chronic kidney disease, while none of the survivors had a history of kidney disease (p value = 0.023). Among the non-survivors, 3 (60.0%) died as a result of severe neurological impairment. One patient developed a large ventricular septal defect after successful weaning from mechanical support and died of acute hemodynamic compromise. Another patient died from hemodynamic instability as a result of Impella device malfunction. 

Clinically relevant adverse events occurred in 6 patients (3 survivors and 3 non-survivors). Four patients developed acute kidney injury; 3 of these patients required temporary renal replacement therapy. Major bleeding requiring transfusion occurred in 3 patients. In 1 patient, severe hemorrhage from the site of Impella implantation required the replacement of the Impella 2.5 with an Impella 5. Two patients also developed bacterial infections during mechanical support. As was mentioned earlier, fatal device malfunction occurred in 1 patient. 

Successful ECMO and Impella weaning was achieved in 5 patients. Two patients were implanted with LV assist devices prior to discharge. The remaining 3 patients were transferred to another hospital to bridge to heart transplantation. No other major complications occurred during the first 28 days following discharge.

**Table 1:** Baseline characteristics of the patients*

Variable	All Patients (n=10)	Survivors (n=5)	Non-Survivors (n=5)	P value
Median age (y)	59 [43-68]	53 [38-59]	64 [58-73]	0.241
Male/Female	7/3	3/4	4/3	0.592
Median BMI, (kg/m^2^)	29 [24-32]	27 [22-31]	30 [27-33]	0.213
Comorbidities				
HTN	7	2	5	0.182
DM	3	1	2	0.412
CAD	7	3	4	0.594
CHF	1	0	1	0.291
CKD	3	0	3	0.028
Baseline EF (%)	20 [18-25]	20 [18-25]	19 [17-25 ]	0.866
Indications for ECMO and Impella				
Cardiac arrest	6	4	2	0.246
Cardiogenic shock	4	1	3	0.157
ECMO duration (days)	6 [4-7]	6 [3-7]	6 [4-8]	0.891

BMI, Body mass index; HTN, Hypertension; DM, Diabetes mellitus; CAD, Coronary artery disease; CHF, Congestive heart failure; CKD, Chronic kidney disease; ECMO, Extracorporeal membrane oxygenation; EF, Ejection fraction

* Data are presented as n or median (interquartile range)

## Discussion

The present study reports the clinical outcomes of Impella implantation in patients on ECMO support. The main finding of our study was a 50% survival rate in patients presenting with cardiac arrest and/or cardiogenic shock, with severe neurologic impairments accounting for 60% of mortality in non-survivors. 

Among the adverse effects of ECMO on a failing heart is the inability to unload the LV. This can cause an increased LV pressure and LV distention, leading to pulmonary edema.^   8 ^ In addition, the stasis associated with inadequate LV unloading could lead to thrombus formation within the LV cavity.^   15 ^ A number of strategies have been suggested to decompress LV distention in patients on ECMO support including intra-aortic balloon pump, percutaneous atrial septostomy, central ECMO cannulation, and the Impella device.^7^^, ^^16^^-^^19^ The Impella device is an attractive alternative in this regard since it is implanted percutaneously without the need for surgical intervention. Also, the forward flow generated by the Impella could prevent potential LV stasis and thrombosis formation. Although only a limited number of studies have evaluated the efficacy of the Impella in patients on ECMO support, the results have been promising.^17^^-^^22^ In the present study, the combination of Impella and ECMO support was able to achieve hemodynamic stability in 9 out of 10 patients. 

Major Impella device malfunction occurred in 1 patient in the present series, which led to severe hemodynamic instability and death. Device malfunction is among the most commonly seen complications of Impella implantation. It has been reported that up to 10% of Impella devices may experience malfunction and cause serious hemodynamic compromise.^23^^, ^^24^ Awareness of this potentially fatal complication as well as intraoperative strategies to prevent cardiovascular collapse is necessary in patients experiencing device malfunction. 

Another major complication seen in the present study was bleeding, which required transfusion in 40% of the patients. As was previously described, the incidence of bleeding is high during venoarterial ECMO support, with some studies reporting up to 100% RBC transfusion requirement.^24^^, ^^25^ The main causes of bleeding include hemolysis and bleeding at the surgical site. 

The main limitations of the present study are its low number of patients and retrospective, single-center nature. In addition, the present study was not a controlled trial with a comparison arm. Therefore, the results of the present study should be interpreted in the context of its limitations. 

## Conclusion

The present study indicates that the application of the Impella device during ECMO support is safe, feasible, and effective in LV unloading and provides optimal hemodynamic support. Further studies are needed to confirm the clinical effectiveness of this approach in larger numbers of patients. 

## Notes:

***This paper should be cited as:*** Moazzami K, Dolmatova EV, Cocke TP, Elmann E, Vaidya P, Ng AF, Satya K, Narayan RL. Left Ventricular Mechanical Support with the Impella during Extracorporeal Membrane Oxygenation. J Teh Univ Heart Ctr 2017;12(1):11-14.
